# Could hemophagocytic lymphohistiocytosis be the core issue of severe COVID-19 cases?

**DOI:** 10.1186/s12916-020-01682-y

**Published:** 2020-07-15

**Authors:** Violetta Opoka-Winiarska, Ewelina Grywalska, Jacek Roliński

**Affiliations:** 1 Department of Paediatric Pulmonology and Rheumatology, Medical University of Lublin, Gębali 6, 20-093 Lublin, Poland; 2 Department of Clinical Immunology and Immunotherapy, Medical University of Lublin, Chodzki 4a Street, 20-093 Lublin, Poland; 3 Department of Clinical Immunology, St. John’s Cancer Hospital, K. Jaczewskiego 7 St, 20–090 Lublin, Poland

**Keywords:** Coronavirus, Coronavirus disease 2019, Hemophagocytic lymphohistiocytosis

## Abstract

**Background:**

COVID-19, a disease caused by the severe acute respiratory syndrome coronavirus 2 (SARS-CoV-2), commonly presents as fever, cough, dyspnea, and myalgia or fatigue. Although the majority of patients with COVID-19 have mild symptoms, some are more prone to serious outcomes, including pneumonia, acute respiratory distress syndrome (ARDS), and even death. Hemophagocytic lymphohistiocytosis (HLH) is a severe, life-threatening inflammatory syndrome associated with intense cytokine release (also known as a “cytokine storm”). Similar to COVID-19, HLH is characterized by aggressive course leading to multi-organ failure.

**Main text:**

The purpose of this review article is to draw attention to the possibility of the complication of HLH in patients with the severe course of COVID-19. Indeed, some of the clinical characteristics observed in the more severe cases of COVID-19 are reminiscent of secondary HLH (which can be triggered by infections, malignancies, rheumatological diseases, or autoimmune/immunodeficiency conditions). The pathogenesis of SARS-CoV-2 infection also suggests that HLH or a similar hyperinflammatory syndrome is the cause of the severe course of the infection.

**Conclusion:**

The pathogenesis and clinical symptoms of severe COVID-19 indicate that an increased inflammatory response corresponding to HLH is occurring. Therefore, patients with severe COVID-19 should be screened for hyperinflammation using standard laboratory tests to identify those for whom immunosuppressive therapy may improve outcomes.

## Background

An acute infectious disease caused by the severe acute respiratory syndrome coronavirus 2 (SARS-CoV-2), named the coronavirus disease 2019 (COVID-19), presents an imminent public health threat worldwide. As of the 23rd of June 2020, over 8.8 million cases of COVID-19 have been confirmed worldwide, and the total number of deaths has surpassed 460,000 [1]. Recent reports have summarized the clinical presentation of COVID-19, which commonly presents as fever, cough, dyspnea, and myalgia or fatigue [2–8]. Although the majority of patients with COVID-19 have mild symptoms, some patients (especially those with underlying diseases) are more prone to serious outcomes, including pneumonia, acute respiratory distress syndrome (ARDS), and even death [9, 10]. Current research efforts are focused on identifying the cause of the aggressive course of the disease and the high mortality rates observed with severe COVID-19, as well as developing novel therapies [10].

Some of the clinical characteristics observed in the more severe cases of COVID-19 [6–8] are reminiscent of hemophagocytic lymphohistiocytosis (HLH), a severe, life-threatening inflammatory syndrome associated with intense cytokine release (also known as a “cytokine storm”) [11]. HLH is characterized by aggressive course leading to multi-organ failure [12]. As HLH can develop in response to viral infections [12], it may be triggered by SARS-CoV-2, which could explain the rapid disease progression observed in some patients.

This review summarizes the pathogenesis and clinical characteristics of COVID-19 that suggests HLH or a similar hyperinflammatory syndrome is the cause of the severe course of the infection. A timely diagnosis of HLH in patients with COVID-19 would offer new therapeutic strategies (e.g., immunosuppression), which in turn, may reduce the significant mortality rates currently associated with this virus.

## Main text

### HLH: an aberrant immune response to viral infections

The majority of viral infections acquired by non-immunosuppressed individuals are asymptomatic or result in mild clinical manifestations; however, for those who are immunocompromised or have an immune disorder, viral infections may result in a life-threatening disease, as occurs in the case of HLH (Table 1) [13]. In HLH, aberrant activation of T cells, natural killer (NK) cells, and macrophages causes overproduction of inflammatory cytokines (i.e., the so-called cytokine storm) and hemophagocytosis [13, 14]. This excessive autoinflammatory response leads to rapidly progressing multi-organ failure [13].

**Table 1:** Effects of immune status on the course of viral infections, outcomes, and therapy

	Normal immunity	Immunodeficiency (primary or secondary)	Immune disorder (genetic or acquired)
**Response to infection**	Correct	Insufficient	Excessive
**Course of viral infection**	Infection limitation and subsequent elimination	Disseminated, systemic or chronic viral infection	Disseminated or systemic inflammation (i.e., HLH, CRS)
**Consequences**	Recovery	Single or multi-organ failure	Multi-organ failure
**Potential interventions**	Vaccinations Antiviral drugs	Vaccinations Antiviral drugs Intravenous immunoglobulins	Immunosuppression

*Abbreviations*: *CRS* cytokine release syndrome, *HLH* hemophagocytic lymphohistiocytosis

HLH is generally divided into two types: primary or familial HLH (which is observed in pediatric patients) and secondary HLH (sHLH, found also in adults). Primary HLH is caused by genetic defects (e.g., mutations in *PRF1* or *UNC13D*, which are typically involved in the perforin-mediated killing of target cells [11]), while a range of triggers are described for sHLH, including infections, malignancies, rheumatological diseases, or autoimmune/immunodeficiency conditions [13, 15]. Epstein-Barr virus (EBV) and herpes simplex virus (HSV) infections are the most frequent triggers of sHLH, although other viruses (e.g., cytomegalovirus, hepatitis A, parvovirus B19, adenovirus, influenza) and pathogens (e.g., bacteria, fungi, parasites) have also been implicated [13–19]. In cases of primary HLH, several different gene defects can lead to the common phenotype of impaired NK/T cell cytotoxicity [20]. Defects in the function of NK and cytotoxic T cells also lead to excessive inflammation in sHLH, when these cells are activated by an external trigger [20].

### The clinical characteristics of COVID-19 resemble sHLH

The cardinal features of sHLH are high fever, hepatomegaly, splenomegaly, cytopenia (e.g., anemia, thrombocytopenia, and neutropenia), coagulopathy, central nervous system disturbances, and rapidly progressing multi-organ failure [14, 16–19]. Respiratory symptoms, which commonly present as dyspnea and cough, or ARDS can also occur in patients with sHLH. This phenomenon mostly occurs in cases triggered by respiratory viruses, and the signs of infection may overlap with the symptoms of sHLH [21]. Similarly, the majority of patients with COVID-19 present with high fever (observed in 44% of patients upon presentation, and subsequently, in 64.5–99% patients), cough (45–82%), dyspnea (6.5–63.5%), and myalgia or fatigue (11–70%) [2–8]. Some patients also show liver damage (transaminase activity), lymphocytopenia, and rapidly progressing multi-organ failure [9, 10, 14, 16, 18]. Indeed, a number of the cardinal clinical features of these two conditions are shared, as summarized in Table 2.

**Table 2:** Comparison of severe coronavirus infection and the symptoms of HLH

	Adult HLH					COVID-19					
Source	Ramos et al. [14]	Zhao et al. [19]	Apodaca et al. [16]	Otrock and Eby [18]	Barba et al. [17]	Huang et al. [4]	Chen et al. [2]	Wang et al. [6]	Zhou et al. [8]	Yang et al. [7]	Spiteri et al. [5]
**Number of patients (%)**	775 (100%)	171 (100%)	64 (100%)	73 (100%)	71 (100%)	41 (100%)	99 (100%)	138 (100%)	191 (100%)	52 critically ill (100%)	31 (100%)
**Clinical symptoms belonging to the HLH criteria** [22]											
**Fever**	524/546 (96%)	171/171 (100%)	63/64 (94.4%)	70/73 (95.9%)	59/71 (92%)	32/41 (78%)	82/99 (83%)	136/138 (99%)	180/191 (94%) ≥ 37.3 °C	51/52 (98%)	20/31 (64.5%)
**Splenomegaly**	420/609 (69%)	146/171 (85.4%)	50/64 (78.1%)	44/73 (60.3%)	27/71 (39%)	No data	No data	No data	No data	No data	No data
**Hemophagocytosis**	257/304 (85%)	152/171 (88.9%)	49/64 (76.6%)	52/68 (76.5%)	57/71 (83%)	No data	No data	No data	No data	No data	No data
**Cytopenias (affecting at least two lineages)**	Yes	Yes	63/64 (98.4%)	62/73 (84.9%)	Yes	No data	No data	No data	No data	No data	No data
Anemia (< 9 g/dL)	122/181 (67%)	98/171 (57.3%)	30/64 (46.9%)	No data	No data	No data	50/99 (51%)	No data	29/191 (15%)	No data	No data
Thrombocytopenia (< 100 × 10^3^/mL)	178/227 (78%)	156/171 (91.2%)	443/64 (67%)	No data	32/71 (45%)	2/41 (5%)	12/99 (12%)	Platelet count of 112–202 × 10^3^/mL	13/191 (7%)	No data	No data
Neutropenia (< 1 × 10^3^/mL)	61/144 (42%)	59/171 (34.5%)	9/64 (14%)	No data	No data	No data	No data	Neutrophil count of 2.0–7.9 × 10^3^/mL	No data	No data	No data
Lymphocytopenia	No data	No data	20/64 (31%)	No data	No data	26/41 (63%)	35/99 (35%)	97/138 (70%)	77/191 (40.3%)	44/52 (85%)	No data
Leukopenia	198/285 (69%)	No data	No data	No data	7/71 (10%)	No data	No data	No data	No data	No data	No data
**Hypertriglyceridemia > 265 mg/dL**	> 265 mg/dL, 42/100 (42%) > 150 mg/dL, 139/192 (69%)	62/171 (36.3%)	33/64 (52%)	49/69 (71%)	No data	No data	No data	No data	No data	No data	No data
**Hyperferritinemia (> 500 ng/mL)**	> 500 ng/mL, 178/198 (90%) > 1000 ng/mL, 164/230 (71%)	165/171 (96.5%)	> 500 ng/mL, 64/64 (100%) > 2000 ng/mL, 49/64 (77.2%)	73/73 (100%)	No data	No data	62/99 (63%)	No data	102/128 (80%) had ferritin > 300 ng/mL	No data	No data
**Elevated sCD25 (soluble IL-2 receptor**)	> 2400 IU/mL, 95/120 (79%)	No data	Yes 64/64 (100%) (inclusion criteria)	24/31 (77.4%)	No data	No data	No data	No data	No data	No data	No data
**Low or absent NK cell activity**	Yes in some patients, but has not yet been standardized	No data	Yes 64/64 (100%) (inclusion criteria)	4/11 (36.4%)	No data	No data	No data	No data	No data	No data	No data
**Hypofibrinogenemia (≤ 150 mg/dL)**		106/171 (62%)	20/64 (32%)	24/64 (37.5%)	No data	No data	No data	No data	No data	No data	No data
**Other symptoms of HLH**											
**Hepatomegaly**	389/580 (67%)	70/171 (40.9%)	46/64 (71.9%)	13/73 (17.8%)	31/71 (44%)	No data	No data	No data	No data	No data	No data
**Pulmonary involvement**	61/145 (42%)	No data	21/64 (32.8%)	No data	ARDS in 44/71 (64%)	ARDS in 12/41 (29%)	ARDS in 17/99 (17%)	ARDS in 27/138 (20%)	ARDS in 59/191 (31%)	ARDS in 35/52 (67%)	No data
**Peripheral adenopathy**	91/277 (33%)	No data	No data	No data	No data	No data	No data	No data	No data	No data	No data
**Neurological symptoms**	41/161 (25%)	No data	10/64 (15.6%)	No data	Confusion or coma in 6/71 (9%)	Headache in 3/38 (8%)	Confusion in 9/99 (9%) Headache in 8/99 (8%)	Headache in 9/138 (6.5%)	No data	Headache in 3/52 (6%)	Headache in 6/31 (19%)
**Multi-organ failure (MOF)/sepsis**	ICU admission in ~ 50% of cases	No data	No data	No data	MOF in 40/71 (56%)	ICU care in 13/38 (32%)	ICU care in 23/99 (23%) Septic shock in 4/99 (4%)	ICU care in 36/138 (26%)	Sepsis in 112/191 (59%) ICU care in 50/191 (26%)	Sepsis in 1/52 (2%)	No data
**Renal insufficiency/failure**	9/56 (16%)	No data	25/64 (39/1%)	38/73 (52.1%)	No data	3/41 (7%)	3/99 (3%)	5/138 (3.6%)	28/191 (15%)	15/52 (29%)	No data
**Elevated CRP**	80–90%	No data	No data	No data	44/71 (62%)	No data	63/73 (86%)	No data	No data	No data	No data
**Elevated serum transaminases**	ALT > 40 IU/L, 164/286 (57%) AST > 100 IU/L, 48/115 (42%)	Yes	47/64 (74%)	61/73 (83.6%)	No data	AST 15/41 (37%)	ALT 28/99 (28%) AST 35/99 (35%)	No (normal levels)	ALT 59/189 (31%)	15/52 (29%)	No data
**Elevated LDH**	> 500 IU/L, 190/243 (78%)	Yes	No data	64/69 (92.8%)	No data	29/40 (73%)	75/99 (76%)	55/138 (40%)	123/184 (67%)	No data	No data
**Elevated D-dimers**	> 54.8 mmol/L 24/49 (49%)	Yes	No data	No data	No data	No data	36/99 (36%)	No (normal levels)	72/172 (42%)	No data	No data
**Elevated serum levels of immunological markers (e.g., IL-2, IL-7, IL-10, G-SCF, IP-10, MCP1, MIP1A, TNF-α)**	Yes	No data	No data	No data	No data	Yes	No data	No data	No data	No data	No data
**Increased IL-6**	Yes	No data	No data	No data	No data	No data	51/99 (52%)	No data	No data	No data	No data

*Abbreviations*: *ALT* alanine aminotransferase, *ARDS* acute respiratory distress syndrome, *AST* aspartate aminotransferase, *CRP* C-reactive protein, *G-CSF* granulocyte-colony stimulating factor, *HLH* hemophagocytic lymphohistiocytosis, *ICU* intensive care unit, *IFN-γ* interferon-γ, *IL* interleukin, *IP-10* interferon-γ-induced protein 10, *LDH* lactate dehydrogenase, *TNF-α* tumor necrosis factor-alpha

In terms of laboratory findings, cytopenia is often observed in sHLH, with thrombocytopenia identified in 80–90% of cases [14, 16, 17, 19]. In addition, almost 60% of patients with HLH have coagulation disorders, while hypofibrinogenemia and raised D-dimer levels are reported in ~ 40–60% of HLH cases [14, 18, 19]. Furthermore, ~ 80% of patients have altered liver test results (i.e., increased phosphatase alkaline and transaminase concentrations), and increased serum lactate dehydrogenase (LDH) concentrations resulting from cell destruction are reported in 78–92.8% of patients [14, 16, 18, 19]. Hypertriglyceridemia (associated with lipoprotein lipase inhibition caused by excess tumor necrosis factor-alpha [TNF-α]) is found in ~ 36–71% of adults with HLH [14, 16, 18, 19]. Increased acute phase reactants (i.e., erythrocyte sedimentation rate or C-reactive protein [CRP] concentration) are identified in 62–90% of patients [14, 17]. Moreover, 90–100% of adult sHLH patients show increased ferritin concentrations (due to increased secretion of ferritin by macrophages or hepatocytes) [14, 16, 18, 19]. Finally, high serum concentrations of soluble CD25 (interleukin [IL]-2 receptor-α) occur in 77–79% of adult cases of sHLH [14, 18], although only very high levels of soluble CD25 are specific to HLH [23]. Other markers of macrophage activation (e.g., β_2_-microglobulin) and cytokines (e.g., interferon [IFN]-γ, TNF-α) are also elevated in HLH [14].

Similar to sHLH, COVID-19 patients present with several laboratory abnormalities, with severe cases showing more prominent abnormalities (i.e., lymphocytopenia, thrombocytopenia, elevated CRP levels) than non-severe cases [24]. Elevated D-dimer, serum ferritin, LDH, and IL-6 levels were also shown throughout the clinical course of non-surviving patients with SARS-CoV-2 pneumonia compared with survivors [8]. In a series of 1449 hospitalized subjects with COVID-19, baseline and maximum values of prothrombin time, activated partial thromboplastin time, and D-dimer levels were significantly higher in subjects who died than in survivors [24]. Subjects who died had higher fibrinogen concentrations at baseline, but lower minimum values, than survivors [24]. Baseline D-dimer levels and the difference in fibrinogen and platelet levels correlated with an increased risk of death among patients with COVID-19 [24]. Indeed, other observations confirm the relationship between coagulation disorders and prognosis [6, 25, 26].

Coagulation disorders are reported in patients with sHLH, frequently with decreased fibrinogen levels, and can result in severe bleeding complications [27]. Indeed, a low fibrinogen level is one of the main HLH diagnostic criteria [22]. Although this process in HLH is not fully explained, the release of proinflammatory cytokines can cause the release of tissue plasminogen activator and the activation of an alternative fibrinolytic pathway in macrophages [27]. These factors can result in severe consumptive coagulopathy, with elevated fibrinogen degradation and decreased fibrinogen levels. Additionally, liver dysfunction may exacerbate coagulopathy [27]. Therefore, the increase in proinflammatory cytokine release in COVID-19 may lead to analogous coagulation disorders in these patients. Indeed, the abovementioned laboratory abnormalities suggest that a hyper-inflammatory reaction is occurring in patients with severe COVID-19.

### Does SARS-CoV-2 trigger a cytokine storm syndrome?

Due to the clinical similarities between severe cases of COVID-19 and sHLH, it has been postulated that SARS-CoV-2 may be a trigger for a cytokine storm syndrome, like sHLH [28]. Indeed, previous studies have shown the poor outcomes of patients severe acute respiratory syndrome (SARS) and Middle East respiratory syndrome (MERS), which are caused by SARS-CoV and MERS-CoV, respectively, are associated with high levels of proinflammatory cytokines (e.g., IL-1β) in the lower respiratory tract and other tissues [29]. The high expression of IL-1β in these tissues further promotes the expression of other proinflammatory cytokines (e.g., TNF-α and IL-6), resulting in a cytokine storm [30]. For example, activation of NF-κB has been shown to contribute to the inflammation induced after SARS-CoV infection [31]. Similarly, SARS-CoV-2 may trigger sHLH or a related inflammatory syndrome in some patients.

A recent retrospective, multicenter study of 150 confirmed COVID-19 cases in Wuhan, China, reported poor outcomes of patients with elevated ferritin and IL-6, suggesting virally driven hyperinflammation may be associated with mortality [32]. Furthermore, Huang et al. recently reported a cytokine profile resembling sHLH (characterized by increased IL-2, IL-7, granulocyte colony-stimulating factor [G-CSF], IFN-γ-induced protein 10 [IP-10], monocyte chemo-attractant protein 1 [MCP-1], macrophage inflammatory protein [MIP] 1-α, and TNF-α) is associated with the severity of COVID-19 [4]. In particular, IL-6 is thought to contribute to the progression of COVID-19 patients to severe ARDS [33]. A more detailed analysis of the literature has uncovered many similarities between the characteristics observed in severe cases COVID-19 infection and sHLH (as summarized in Table 2). For example, serum ferritin and CRP levels are above the normal range (i.e., in 63–80% and 61–86% of patients, respectively) in patients with severe COVID-19 infection, which is also observed in sHLH [2]. Furthermore, patients with severe COVID-19 infections have been shown to rapidly develop a number of complications, which resemble the multi-organ failure that arises in HLH.

### Severe COVID-19 shows rapid progression similar to HLH

A characteristic feature of severe COVID-19 is that disease progresses rapidly, and the patient develops multi-organ failure in a short period of time [2]. As is observed in cases of HLH [14, 16, 17, 19], patients with severe COVID-19 show rapid signs of multi-organ damage. For example, among 99 patients diagnosed with SARS-CoV-2 pneumonia, 17% developed ARDS, 8% developed acute respiratory injury, 3% developed acute renal injury, and 4% progressed to septic shock [2]. In addition, among 52 critically ill patients with SARS-CoV-2 pneumonia, 67% had ARDS, 29% had acute renal injury, 23% had cardiac injury, 29% had liver dysfunction, and 2% had pneumothorax [7]. In another retrospective, single-center case series of 138 consecutive hospitalized patients with confirmed SARS-CoV-2 pneumonia, 8.7% developed septic shock, 19.6% developed ARDS, 16.7% had arrhythmias, and 7.2% had acute cardiac injury [6]. In a retrospective, multi-center cohort including 191 adult inpatients with laboratory-confirmed COVID-19, sepsis was the most frequently observed complication (observed in 59% of cases), followed by respiratory failure (54%), ARDS (31%), heart failure (23%), and then septic shock (20%) [8]. In terms of the times of onset for the various complications arising from COVID-19, sepsis is reported to develop a median of 9 days after illness onset, followed by ARDS (12 days), acute cardiac injury (15 days), acute renal injury (15 days), and then secondary infection (17 days) [8]. As COVID-19 follows a similar pathogenesis to sHLH, early diagnosis and prompt immunosuppression is key, before such multi-organ failure sets in [34].

### Diagnosing HLH in patients with COVID-19

**Fig. 1:**
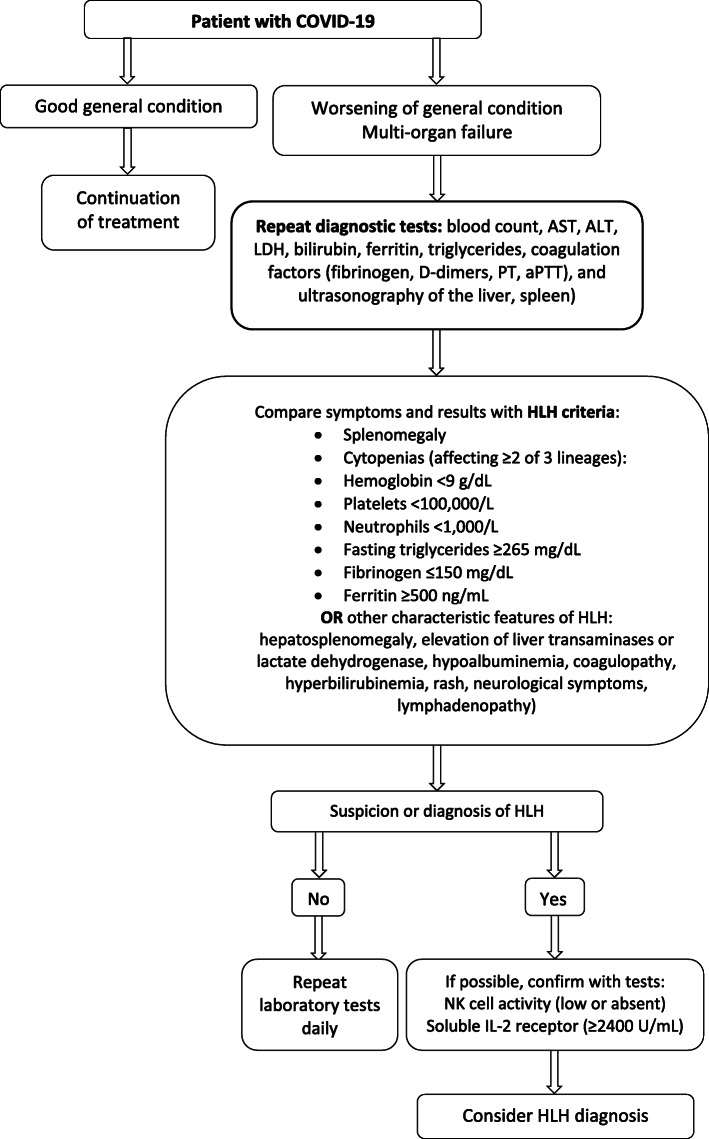
Proposed diagnostic scheme for patients with COVID-19. Abbreviations: ALT, alanine aminotransferase; AST, aspartate aminotransferase; COVID-19, coronavirus disease 2019; HLH, hemophagocytic lymphohistiocytosis; IL-2, interleukin-2; LDH, lactate dehydrogenase; NK, natural killer; PT, prothrombin time; aPPT, activated partial thromboplastin time

The diagnosis of sHLH is based on clinical symptoms and results of diagnostic tests. According to the revised HLH-2004 guideline [35], which was recently updated for adult patients [36], the diagnosis is based on five criteria (fever, splenomegaly, bicytopenia, hypertriglyceridemia and/or hypofibrinogenemia, and hemophagocytosis) and three additional criteria: low/absent NK-cell-activity, hyperferritinemia, and high-soluble IL-2-receptor levels. Other abnormal clinical and laboratory findings consistent with the diagnosis are cerebromeningeal symptoms, lymph node enlargement, jaundice, edema, skin rash, hepatic enzyme abnormalities, hypoproteinemia, hyponatremia, VLDL increase, and HDL decrease. Five of these eight criteria must be fulfilled, unless family history or molecular diagnosis is consistent with HLH. Absence of hemophagocytosis does not exclude a diagnosis [35]. Nonetheless, a simple score for the diagnosis of HLH is freely available online, named the Hscore [37]. The problem is that these criteria mainly correspond to primary HLH, not always to sHLH [38]. For example, the macrophage activation syndrome (MAS) – sHLH associated with autoimmune diseases is diagnosed based on other criteria: i.e., thrombocytopenia, hypofibrinogenemia, hypertriglyceridemia with other cut-off values, and high aspartate aminotransferase (AST) levels, which are included in the HLH-2004 criteria [38]. Therefore, it is likely that a different set of criteria would be needed to diagnose sHLH associated with COVID-19 [39]. Nevertheless, based on current evidence, sHLH should be suspected in patients with worsening or severe COVID-19, and early diagnosis could potentially be made using a panel of diagnostic tests based on the Hscore (see Fig. 1).

It is also important to mention that HLH presents with similar characteristics to other inflammatory disorders, such as sepsis, septic shock, and cytokine release syndrome (CRS) [22]. Sepsis is a life-threatening organ dysfunction caused by a dysregulated host response to infection and is diagnosed according to a suspected or documented infection and an acute increase of ≥ 2 SOFA (Sequential [Sepsis-Related] Organ Failure Assessment Score) points (a proxy for organ dysfunction [38]). Septic shock is a subset of sepsis, in which underlying circulatory and cellular or metabolic abnormalities substantially increase mortality.

Unfortunately, the current criteria do not allow a clear differentiation of sepsis from HLH, and it is proposed that sepsis and HLH may have a common mechanism, whereby systemic immune dysregulation is triggered by a specific external agent [14]. CRS is another systemic inflammatory response that can be triggered by infections and can present with similar symptoms (e.g., fever, fatigue, headache, rash, arthralgia, myalgia, uncontrolled systemic inflammatory response, and multi-organ failure) and laboratory abnormalities (e.g., cytopenias, elevated creatinine and liver enzymes, abnormal coagulation parameters, and high CRP levels) to HLH [40]. Respiratory symptoms are also common in patients with CRS, including ARDS, as well as renal failure or cardiac dysfunction [40]. Therefore, the diagnosis of HLH is complicated due to the non-specific clinical manifestations and laboratory findings associated with this condition [15], and more precise criteria should be developed in the future.

### How can an early diagnosis of HLH help in the management of COVID-19?

To date, no effective clinical management has been established for COVID-19 and there is no evidence for specific drug treatment against SARS-Cov-2 in suspected or confirmed cases [36].

For diagnosis and ongoing management of COVID-19, lung imaging (X-ray, computed tomography) and laboratory tests are recommended [41]. Laboratory tests include a throat swab or other respiratory sampling to identify SARS-CoV-2 RNA by PCR; hematology examination (blood count, lymphocyte subpopulation); tests for common respiratory viruses, mycoplasma, chlamydia, and tuberculosis; liver and renal function tests; myocardial enzyme and myoglobin levels; erythrocyte sedimentation rate; CRP, procalcitonin, lactate, and D-dimer levels; coagulation image; a routine urine test; measurement of inflammatory factors (IL-6, IL-10, TNF-α), complement; and anti-acid staining [41] These parameters should be constantly monitored in patients with COVID-19. Adding ferritin, fibrinogen, triglycerides, total protein/albumin, and lactate dehydrogenase to laboratory tests would allow early identification of patients with a cytokine storm syndrome like sHLH.

Effective management of COVID-19 would require either prevention (i.e., a vaccine) or, in the case of infection, specific antiviral treatments and inhibitors of generalized inflammation. Moreover, whether treating sHLH in the course of COVID-19 improves patients outcomes requires further study. Nonetheless, if a diagnosis of sHLH were to be made in patients with COVID-19, it would be beneficial to control the hyperinflammatory reaction that leads to multi-organ failure and death. Although HLH management is based on the HLH-2004 guidelines [22] (which were recently updated for adult patients [36]), the treatment should be modified based on the underlying cause and course of the disease [42]. It is certain that the effectiveness of the therapy is time-dependent; therefore, HLH therapy should be started as soon as possible, preferably on the day of diagnosis [22]. The aim of the initial treatment is to control the hyperactivated immune system. A corticosteroid is usually selected as the first-line treatment, preferably dexamethasone. However, in cases of infection-associated HLH, a high-dose intravenous immunoglobulin (IVIG) is often used for the initial treatment, plasma exchange or exchange transfusion may also be performed to eliminate cytokines and improve the coagulation state [22, 42]. Despite these chemoimmunotherapy recommendations, in EBV-associated HLH (EBV-HLH), some patients may be cured with corticosteroid treatment alone [43]. Furthermore, patients with an infection-associated HLH other than EBV-HLH often enter remission when they are treated with corticosteroids, IVIG, and/or cyclosporine in addition to the treatment for the infectious disease [42]. However, once again, the effectiveness of this treatment relies on the early inclusion of treatment. Therefore, we propose patients with worsening or severe COVID-19 should undergo a diagnostic panel of tests (shown in Fig. 1) and constant monitoring to enable rapid intervention of appropriate treatment.

### Controlling the COVID-19 cytokine storm: experimental therapies

In addition to the HLH-2004 protocol, an anti–IL-6 antibody (tocilizumab) was FDA-approved in 2014 for HLH patients aged ≥ 2 years, as it results in rapid resolution of cytokine release syndrome in patients after chimeric antigen receptor (CAR) T cell or blinatumomab treatment [36]. In 2018, a CAR T cell-associated toxicity working group suggested suspected HLH should be managed with anti-IL-6 therapy as well as corticosteroids for those with organ toxicities ≥ grade 3 [36, 44]. Encouragingly, the recently announced COVACTA trial aims to evaluate the safety and efficacy of intravenous tocilizumab in hospitalized adult patients with severe COVID-19 pneumonia (ClinicalTrials.gov Identifier: NCT04320615) [45], and a multicenter, randomized controlled trial of tocilizumab has been approved in patients with COVID-19 pneumonia and elevated IL-6 2 in China (Chinese Clinical Trial Registry: ChiCTR2000029765) [46]. In addition, IL-1 blockade with anakinra has shown a significant survival benefit in patients with hyperinflammation [47]. Thus, a clinical study to evaluate the efficacy and safety of anakinra and emapalumab (an anti-IFN-γ antibody that is FDA-approved for adult and pediatric patients with primary HLH) in the treatment of hyperinflammatory syndrome associated with severe cases of COVID-19 is currently underway (ClinicalTrials.gov Identifier: NCT04324021) [48].

Janus kinase (JAK) inhibition is another therapeutic strategy, which could affect both inflammation and cellular viral entry in cases of COVID-19 [49]. Activation of the NF-κB (nuclear factor kappa B) signaling pathway was also shown to contribute to the inflammation induced after SARS-CoV-1 infection [31]; therefore, NF-κB inhibitors may be promising for the treatment of severe COVID-19. Thus, there are a number of exciting new therapies in the pipeline to combat severe cases of COVID-19.

## Conclusion

SARS-CoV-2 is also a novel human pathogen that may interact with host antiviral defense in a unique manner. Severe cases of COVID-19 share a number of clinical characteristics with HLH. Without early diagnosis and prompt appropriate treatment, the mortality rate of HLH is very high [13]. Therefore, it is recommended all patients with severe COVID-19 should be screened for hyperinflammation using standard laboratory tests and the HScore [35] to identify the subgroups of patients for whom immunosuppressive therapy may improve outcomes. We acknowledge that a different set of criteria may be required to diagnose patients with COVID-19-associated HLH [39]. Management by a multidisciplinary team of experts (including hemato-oncologists, immunologists, rheumatologists, and intensivists) will be required to provide patients with access to such a full range of treatment options.

## Data Availability

Not applicable

## Abbreviations

ALT: Alanine aminotransferase
AST: Aspartate aminotransferase
ARDS: Acute respiratory distress syndrome
COVID-19: Coronavirus disease 2019
CRP: C-reactive protein
CRS: Cytokine release syndrome
G-CS: Granulocyte-colony stimulating factor
HLH: Hemophagocytic lymphohistiocytosis
Hscore: Score for the diagnosis of HLH
IFN-γ: Interferon-γ
IL: Interleukin
IP-10: Interferon-γ-induced protein 10
JAK: Janus kinase
LDH: Lactate dehydrogenase
MAS: Macrophage activation syndrome
MERS: Middle East respiratory syndrome
MCP-1: Monocyte chemo-attractant protein 1
MIP: Macrophage inflammatory protein
NK: Natural killer
NF-κB: Nuclear factor kappa B
PT: Prothrombin time
aPPT: Activated partial thromboplastin time
SARS: Severe acute respiratory syndrome
SARS-CoV-2: Severe acute respiratory syndrome coronavirus 2
sHLH: Secondary hemophagocytic lymphohistiocytosis
SOFA: Sequential [Sepsis-Related] Organ Failure Assessment Score
TNF-α: Tumor necrosis factor-alpha
